# Postoperative pain after endodontic treatment of necrotic teeth submitted to large apical preparation using oscillatory kinematics

**DOI:** 10.4317/jced.58726

**Published:** 2022-02-01

**Authors:** Ricardo Machado, Daniel Comparin, Sérgio-Aparecido Ignácio, Ulisses-Xavier-da Silva Neto

**Affiliations:** 1 DDS, MSc, PhD. Department of Endodontics, School of Health and Bioscience, Pontifical Catholic University of Paraná - PUC/PR, Curitiba, Paraná, Brazil; 2 DDS, MSc. Department of Endodontics, School of Dentistry, Paranaense University - UNIPAR, Francisco Beltrão, Paraná, Brazil; 3 DDS, MSc, PhD. Department of Statistics, School of Health and Bioscience, Pontifical Catholic University of Paraná - PUC/PR, Curitiba, Paraná, Brazil

## Abstract

**Background:**

Regardless of the technique applied for chemomechanical preparation, postoperative pain is a very relevant matter in endodontics. Objective: To evaluate postoperative pain after endodontic treatment of necrotic teeth submitted to large apical preparation (LAP) using oscillatory kinematics.

**Material and Methods:**

The sample included 60 asymptomatic necrotic teeth with or without apical radiolucency, and with normal periodontal status, referred for endodontic treatment. Following initial procedures, the position and approximate size of the apical constriction were determined by using an apex locator and K Flexofiles, respectively. The chemomechanical preparation was conducted using oscillatory kinematics and 2.5ml of 2.5% NaOCl at each file change to achieve LAP, and the filling was done with Tagger’s hybrid technique and EndoFill sealer. Phone calls were made to all the patients at 24, 48 and 72 hours after treatment to request their classification of postoperative pain, and data were submitted to statistical analysis.

**Results:**

Only 3 patients (5%) reported severe pain after 72 hours. Moderate pain was reported by 17, 9 and 1 patient after 24, 48 and 72 hours, respectively (*P* = 0.000). However, paired analyses showed a statistically significant difference only between 24 and 72 hours (*P* = 0.001), and 48 and 72 hours (*P* = 0.014). Age and tooth type did not influence the postoperative pain, regardless of time (*P*> 0.05). After 72 hours, women experienced significantly more pain than men (*P* = 0.012), and teeth without periradicular lesion were more sensitive that teeth with perirradicular lesion (*P* = 0.027).

**Conclusions:**

Acute or moderate postoperative pain was uncommon after endodontic treatment of necrotic teeth submitted to LAP using oscillatory kinematics.

** Key words:**Endodontic treatment, oscillatory kinematics, postoperative pain, pulp necrosis.

## Introduction

Endodontic treatment aims to maintain or reestablish the health of periapical tissues by cleaning and filling the root canal system (RCS) (1,2). Chemomechanical preparation is unable to eliminate the entire content of the RCS, mostly because of its anatomical and morphological features (3). Nonetheless, combined use of endodontic instruments and chemical solutions, associated with the physical cleaning action promoted by irrigation (flow and counterflow of a liquid under pressure), represents the main responsible for root canal disinfection (4).

The amplitude of apical root canal instrumentation is a controversial issue in endodontics (5,6). The established postulation is that dentinal wear must be sufficient to provide good cleaning and disinfection, without making the endodontically treated tooth too fragile, and ultimately susceptible to premature loss (7). However, there are clear indicators that would recommend larger apical preparation (LAP) (8,9).

The introduction of nickel-titanium (NiTi) instruments has allowed safer and easier chemomechanical preparations to be performed, even in cases of anatomical complexity (10). The rotary and reciprocating techniques have improved significantly in the past few years, especially with the development of new file types. However, considering that NiTi file separate in two distinct modes – torsional and flexural (11,12) – the risk of separation when performing LAP with these kinds of instruments becomes imminent because its resistance to fracture decreases with increasing instrument diameters, specifically with core dimensions (11,13).

The oscillatory mechanical systems, such as M4 (Kerr, SybronEndo, Orange, CA), 3LD (KaVo, Biberach Germany), TEP E-10 and 16R (NSK Nakanishi, Tochigi-ken, Japan), are boosted by electric or pneumatic motors, and has the advantage of being used with both stainless steel and NiTi hand files (14). When correctly performed, the oscillatory technique is effective (15), inexpensive (16) and safe for conducting LAP, even in curved canals (17).

Regardless of the technique applied for chemomechanical preparation, postoperative pain is a very relevant matter in endodontics. Some important points associated with this negative consequence include bacterial remnants in the RCS, and mechanical, chemical and infectious trauma to periradicular tissues (18,19). Recent studies have reported controversial results regarding the extrusion of debris after root canal shaping using different techniques (20-22).

Considering that there is a direct relationship between extrusion of debris and postoperative pain, and that no study has yet been performed to evaluate the latter after conducting endodontic treatment of necrotic teeth submitted to LAP using oscillatory kinematics, the current research was conducted to achieve this objective.

## Material and Methods

This study received the approval of the Human Research Ethics Committee of the Pontifical Catholic University of Paraná - PUC/PR, Curitiba, Paraná, Brazil (CAAE. 99497118.6.0000.0020), and was reported in accordance with the Consolidated Standards of Reporting Trials Statement (CONSORT, 2010).

-Sample size calculation

The sample size to validate the results of this research was determined through a pilot study showing that less than 5% of the patients reported significant postoperative pain (acute or moderate) after treatment. The proportion-sampling method was used to determine the sample size, which was set at 60 teeth, considering a confidence level of 95%, and a maximum margin of error of 5.5% (23).

-Case selection

This study was conducted on patients ranging in age from 16 to 80 years, referred to the Centro de Especialidades Odontológicas de Navegantes - CEO, Navegantes, Santa Catarina, Brazil, for endodontic treatment between September and October 2019. The inclusion criteria were necrotic teeth with or without asymptomatic apical periodontitis, and with a periodontal probing depth of 3 mm at most, accessed previously at the Public Basic Health Units of the aforementioned city. The exclusion criteria were consumption of anti-inflammatories, analgesics of any kind, or antibiotics within the last 10 days before treatment, presence of root resorption, sinus tracts, trismus, periodontal probing greater than 3 mm, systemic diseases, history of trauma, pregnancy, severe malocclusion associated with traumatic occlusion, lack of patient compliance, and history of intolerance to nonsteroidal anti-inflammatory drugs.

-Treatment protocol

After the health status of the patients was evaluated, followed by clinical and radiographic examinations of their teeth, the patients were informed of the available alternative treatment options and the postoperative care that needed to be done. Information on the study and the endodontic treatment protocol were also provided to all the patients or their caregivers (for patients under 18 years old), and written consent was obtained. First, the teeth were anesthetized using 2% mepivacaine with epinephrine 1:100.000 (Mepiadre; DFL Indústria e Comércio, Rio de Janeiro, RJ, Brazil). After rubber dam placement and disinfection, the temporary restoration was removed using a 1014 or 1016 HL bur (KG Sorensen, Barueri, SP, Brazil). When reaching the pulp chamber, copious irrigation was performed with 5 mL 2.5% sodium hypochlorite (NaOCl) (Fórmula & Ação, São Paulo, SP, Brazil), and the canals were flooded continuously with irrigation solution from a NaviTip 31 G needle (Ultradent, South Jordan, UT, USA). An initial exploration was conducted with a manual stainless-steel #15 K Flexofile (Dentsply-Maillefer, Ballaigues, Switzerland). Cervical and middle thirds were prepared with #1, 2, 3 or 4 Gates-Glidden drills (Dentsply-Maillefer), depending on the anatomical features of each tooth. Afterwards, the position of the apical foramen was established by #10 or larger manual, stainless-steel K Flexofiles (Dentsply-Maillefer), coupled to an apex locator (Root ZX II, J Morita, Kyoto, Japan), and confirmed by radiographs. The working length (WL) was established 1 mm short of this length (near the apical constriction) (24). The approximate size of this anatomical structure, i.e. the anatomical diameter (25,26), was determined by using #10, 15, 20, 25 or 30 manual stainless-steel K Flexofiles (Dentsply-Maillefer). This strategy was crucial to the instrumentation planning of similar LAPs for treating different teeth.

Chemomechanical preparation was performed by manual stainless-steel K files and K- Flexofiles (Dentsply-Maillefer) attached to the handpiece of a TEP E-16R oscillatory system driven by a pneumatic engine (Kavo do Brasil, Joinville, SC, Brazil), using a crown-down approach. A 2.5 mL aliquot of 2.5% NaOCl (Fórmula & Ação) was used as an irrigating solution at each file change, applied with a NaviTip 31 G (Ultradent) needle up to 5 mm short of the apical constriction, as established by rubber stops. All the teeth received the same amount of irrigant. LAPs were performed by using 6 files larger than the corresponding file of the anatomical diameter.

After instrumentation, the canals were flooded with 3 mL of 17% EDTA (Fórmula & Ação) for 3 minutes. A final flush was made with 5 mL of saline solution, and the canals were dried with absorbent paper points (Tanari, Manaus, AM, Brazil). A master cone was introduced into each canal, corresponding to the last instrument used during chemomechanical preparation, stabilized to approximately 1 mm from the apical foramen to avoid overextension. After radiographic analysis of the level of the master cone, the root canal filling was performed using Tagger’s hybrid technique and Endofill sealer (Dentsply-Maillefer), and the temporary restoration with Cavitec (Caitech, São José dos *Pi*nhais, PR, Brazil) or Cimpat (Septodont, São Paulo, SP, Brazil). A final radiograph was taken after occlusal adjustment, and the patients were referred back to the Public Basic Health Unit of origin to receive the definitive restoration. No medication was prescribed, and the patients were instructed to take either paracetamol (750 mg every 6 hours) or ibuprofen (600 mg every 6 hours) if they felt significative pain (19). All the treatments were performed by a single, experienced operator (R.M.).

-Analysis of postoperative pain and statistical analysis

Postoperative pain was assessed at 24, 48 and 72 hours after treatment, as outlined below. All the patients were called by phone to inquire into their pain. The postoperative pain was classified according to a score based on a verbal categorization scale (Table 1) (27,28).

**Table 1:**
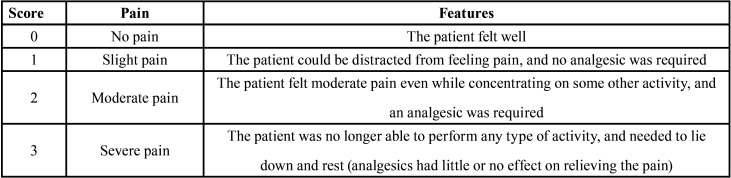
Scores and features of the pain.

The findings were recorded on an Excel spreadsheet (Microsoft Corporation, Redmond, WA, USA) for statistical evaluation with SPSS software version 25.0 (SPSS, Chicago, IL, USA). Kolmogorov-Smirnov’s test was used to evaluate data normality. Mann-Whitney U, Friedman’s ANOVA and Friedman’s multiple 2 to 2 comparison tests were used to determine any significant difference among the variables (*P* < 0.05).

## Results

**Figure 1:**
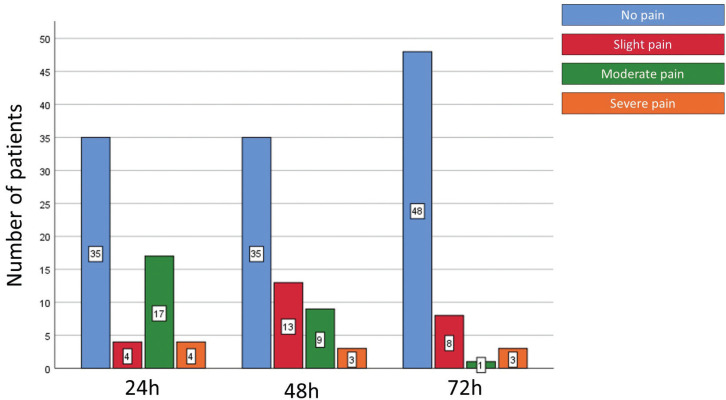
Incidence and levels of postoperative pain after 24, 48 and 72 hours.

The demographic data are shown in Table 2. General incidence and levels of postoperative pain at the study time points were low, and are shown in Figure 1. The postoperative pain tended to decrease over time (*P* = 0.000). However, paired analyses showed a statistically significant difference only between 24 and 72 hours (*P* = 0.001), and 48 and 72 hours (*P* = 0.014). Age and tooth type did not influence the postoperative pain, regardless of time (*P* > 0.05). After 72 hours, women experienced significantly more pain than men (*P* = 0.012), and teeth without periradicular lesion were more sensitive that teeth with perirradicular lesion (*P* = 0.027) (Table 3,  Table 3 cont.).

**Table 2:**
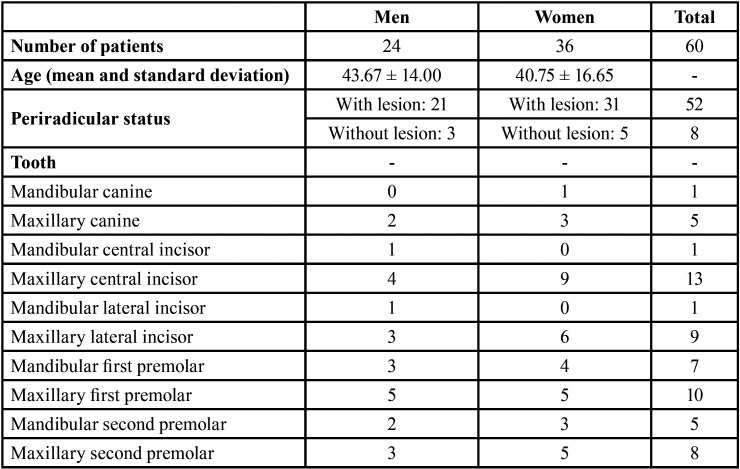
Demographic data.

**Table 3:**
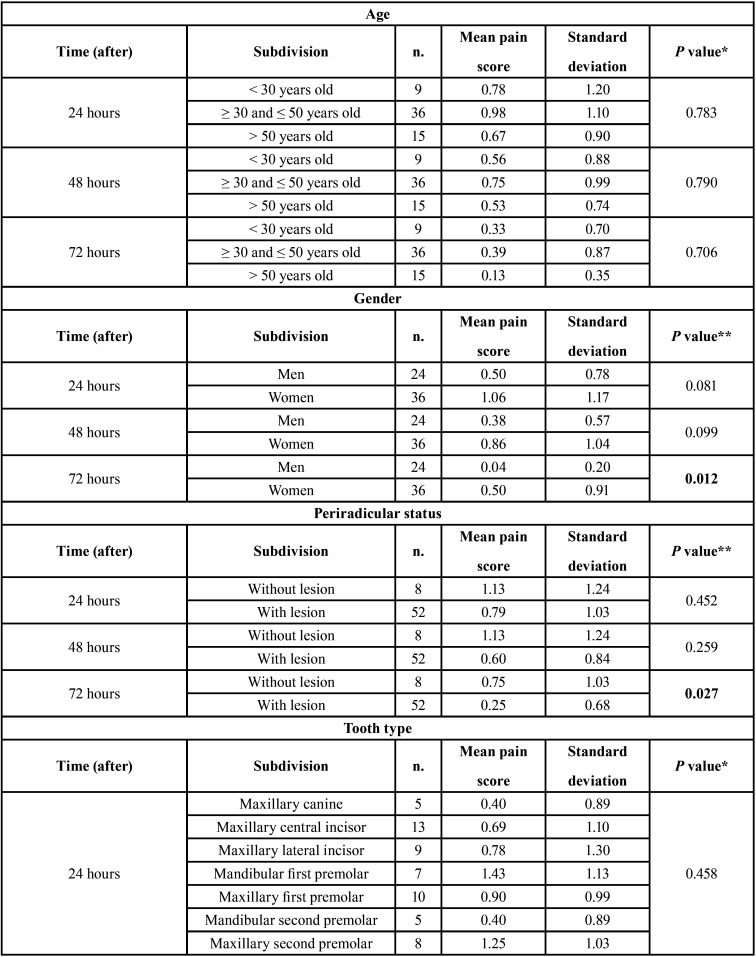
Postoperative pain, considering time point, age, gender, periradicular status, tooth type and time frame.

**Table 3 cont.**
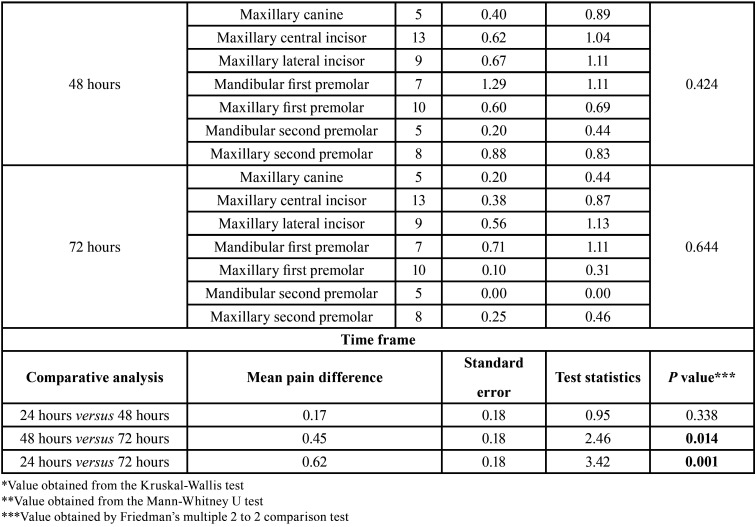
Postoperative pain, considering time point, age, gender, periradicular status, tooth type and time frame.

## Discussion

Postoperative pain in endodontics is a relatively frequent event and is a topic of great interest to both clinicians and researchers. It may occur between sessions or after endodontic treatment has been concluded and is mainly attributed to periradicular tissue injuries of chemical, mechanical or infectious cause. The factors responsible for the incidence and severity of postoperative pain in endodontics have not yet been fully elucidated, owing to the limitations inherent to the applied research. In general, pain is influenced by several factors, in addition to the independent variables under investigation, which are impossible to eliminate in a clinical setting. Another important limitation is that pain is a highly subjective and personal sensation. Nevertheless, there are several well-designed studies able to provide valuable information on this subject (29,30). The aim of the current study was to evaluate postoperative pain after endodontic treatment of necrotic teeth with or without asymptomatic apical periodontitis submitted to LAP performed by oscillatory kinematics.

The factors that act at different time periods in relation to the endodontic treatment (before, during and after, i.e., pre-, intra- and posttreatment) can influence postoperative pain, and can therefore act as confounding factors. Age, gender, tooth type and preoperative pain are considered pre-treatment confounding factors. The number of sessions, the irrigating solutions or intracanal dressing used, the chemomechanical preparation technique, and the sealer represent the intra-treatment confounding factors, whereas the analgesic intake after the treatment represents the posttreatment confounding factor (29,31-34). The establishment of specific eligibility criteria, and the treatment protocols and randomization adopted in the division of patients into groups (if applicable) are essential procedures for minimizing the effect of confounding factors and are therefore crucial for obtaining reliable results (32,35). The widely known assumption that preoperative pain is a strong predictor of postoperative pain (36,37) led the authors to include only symptom-free patients in this study. Single-visit endodontic treatments have been associated with the same postoperative pain (38,39) and healing of periapical tissues (40) as experienced in two-visit treatments. Therefore, all the treatments were performed in a single visit, to minimize the number of procedures, and confounding factors (19,38,41). However, endodontic accesses had already been performed in previous appointments. Lastly, the possibility that operator experience could influence the incidence of postoperative pain (42) led to all treatments being performed by the same operator (R.M.) (19,41,43).

The size of apical preparation is crucial for achieving adequate RCS cleaning and disinfection (5,6). Weine *et al*. (25), reported that the master apical file (MAF) size should enlarge the apical third of the root canal to 3 sizes larger than the first file that bound at the WL, after coronal flaring. However, it is clinically impossible to know where this binding really occurs, and/or whether it actually reflects the anatomical diameter of the root canal (6,26). According to Wu *et al*. (26), the first file to bind in the apical third of the root canal does not necessarily reflect the true apical diameter at the WL, because of anatomical complexity. Therefore, preparing the apical third of the root canal to only 3 sizes larger than the first binding file does not ensure removal of the inner layer of dentin from all apical root canal walls, or all infected necrotic pulp tissue (26). Other authors have suggested that taper is more relevant in instrumentation than the final apical size of the canal, given that a taper size of 0.10 has previously enabled similar results for cleaning the apical third of the root canal when the apical preparation size was 20 or 40 (44). Roças *et al*. (45) showed that no significant bacterial reduction in infected canals was found between NiTi hand (.02 taper) and rotary (.04 taper) files after chemomechanical preparation. Nevertheless, the latter showed more negative results in the same quantitative analysis made by real-time PCR. In that study, the canals were instrumented to a similar apical size. However, Siqueira *et al*. (46) reported that canal preparation up to a #30 NiTiFlex file (.02 taper) (Dentsply-Maillefer) was significantly more effective than a #20 GT file (taper .12) (Dentsply-Maillefer) in reducing intracanal bacteria. Thus, it can be inferred that both taper and diameter are equally important in chemomechanical preparation of the RCS. However, larger tapers have been reported to weaken endodontically treated teeth. Therefore, we opted for performing LAP with manual stainless-steel K files and K Flexofiles (taper .02) in oscillatory motion in this research. Molars and other teeth with curved canals were excluded from the sample to avoid risking apical transportation and root perforation (47).

A systematic review and meta-analysis carried out by Caviedes-Bucheli *et al*. (48) investigated the influence of the number of files (2 full-sequence rotary files systems – ProTaper Universal/Dentsply-Maillefer and Mtwo/VDW, Munich, Germany, versus 2 single reciprocating files systems – WaveOne/Dentsply-Maillefer and Reciproc/VDW) on the extrusion of debris and on the prevalence of symptomatic apical periodontitis. According to the results of laboratory studies, greater extrusion of debris was observed when using single file reciprocating systems compared with full-sequence rotary file systems. Moreover, *in vivo* studies showed that the system design had a greater impact on the expression of neuropeptides in the periodontal ligament than the number of files (49,50). All instrumentation systems cause apical extrusion of debris and the expression of neuropeptides in the periodontal ligament. This supports the hypothesis that the inflammatory reaction is not influenced by the number of instruments, but by the kinematics and design of the files used. Thus, it follows that reciprocating kinematics can provide a more comforTable postoperative condition, as shown in a randomized clinical trial performed by Pasqualini *et al*. (51). Considering the similarities between oscillatory and reciprocating kinematics, our results are in line with those of these studies.

Different methods have been used to assess pain after endodontic therapy, such as visual analog scales (VAS) (19,52), scores based on verbal categorization (53,54), or both (55,56). Farzaneh *et al*. (55) performed a triple-blind randomized clinical trial to compare the effect of two different NaOCl concentrations on postoperative pain and used both 2- and 4-level pain classification systems. It was found that different systems had no influence on the results at different time intervals after treatment. Similar results were found by Attar *et al*. (56), who reported a high correlation between different questionnaires and the classification system used for recording postoperative endodontic pain. Regardless of the classification method, what is most important is to make sure that the questions in a questionnaire are fully understood by the patients and easily interpreted by the researchers (27,57). In the present study, a scoring system was used to classify postoperative pain, based on verbal categorization, as follows: no, slight, moderate, and severe pain. These categories were understood straightforwardly by patients. A similar strategy was used in other studies (27,53,54).

Overall, mean pain scores were low, and just 3 patients (5%) reported acute pain after 72 hours. Similar results were obtained by Relvas *et al*. (53), in the group where the chemomechanical preparation was performed by a rotary (ProTaper Universal, Dentsply-Maillefer) and a reciprocating (Reciproc, VDW) system. We believe that the low incidence and levels of postoperative pain observed in our study are probably related to the same reasons found by Relvas *et al*. (53), namely: a) only teeth with asymptomatic pulp necrosis were treated; b) all teeth suffered occlusal adjustment after the treatment; and, c) in regard to the irrigation protocol, the same volume of irrigant was used, and the needle was inserted at a distance far enough from the apex to prevent extrusion.

Moderate pain was reported by 17, 9 and 1 patient after 24, 48 and 72 hours, respectively, implying that pain tended to decrease over time (*P* = 0.000). However, paired analyses showed a statistically significant difference only between 24 and 72 hours (*P* = 0.001), and 48 and 72 hours (*P* = 0.014). These results are in line with those obtained by a prospective, randomized, double-blind clinical trial performed by Shokraneh *et al*. (58), and a systematic review and meta-analysis conducted by Pak and White (59). Nonetheless, Yaylali *et al*. (41) observed more pain in the 48-hour posttreatment period. This contradictory result may be attributed to the different methodological designs of the studies. In the study by Yaylali *et al*. (41), only necrotic molars with radiographically visualized periradicular lesions were treated. Chemomechanical preparation was performed by using ProTaper Next files after establishing the WL at the apical foramen. The irrigation consisted of 2.5% NaOCl using a Max-i-Probe needle up to 2 mm short of the WL, and the postoperative pain was evaluated by VAS. In the current research, the WL was determined 1 mm short of the apical foramen, chemomechanical preparation was performed with manual stainless-steel K files and K Flexofiles in oscillatory motion, irrigation was conducted with 2.5% NaOCl using a NaviTip needle up to 5 mm short of the apical constriction, and the postoperative pain was evaluated with a score based on verbal categorization.

Our results indicated that age did not influence postoperative pain, thus corroborating the findings by Ng *et al*. (33), and Polycarpou *et al*. (60). On the other hand, Ali *et al*. (61) showed greater postoperative pain in older (41 to 65 years) compared with younger (15 to 40 years) patients. The probable reasons could be lower pain tolerance, less blood flow, and delayed healing (61). However, a direct comparation between our results and those obtained by the 3 previously cited studies (33,60,61) should be made with caution. The age difference between the upper and lower age group limits of the cited studies was 10 years (33,60) and 25 years (61). Herein, the limit was about 20 years. In addition, there was sizable discrepancy in the number of patients in the different study groups. In the study by Ng *et al*. (33) for example, 119 (28.7%) of the 415 patients were ≥ 40 and < 50 years old, and 8 (1.9%) were ≥ 80 years old. In the present study, 9 (15%) of the 60 patients were < 30 years old, 36 (60%) were ≥ 30 and ≤ 50 years old, and 15 (25%) were ˃ 50 years old.

Several studies have shown a higher prevalence and/or duration of postoperative pain in females than males (31,61). In the present study, it was proven once again. After 72 hours, women experienced significantly more pain than men (*P* = 0.012). The biological difference between men and women could explain why (62,63). Moreover, comparing the variation between male and female reproductive and pelvic anatomies, the latter gender has an additional portal of entry of infection, thereby leading to possible local and distant hyperalgesia. In addition, fluctuating female hormonal levels might be associated with changing levels of serotonin and noradrenaline, ultimately leading to increased pain prevalence in females during the menstrual period, or when receiving hormonal replacement therapy or taking oral contraceptives (31,33,61).

The nature of the pulp and periapical status could modulate postoperative pain in endodontics. Teeth with nonvital pulps associated with periapical lesions are densely contaminated, and have a 83.2% prevalence of foraminal resorption (64), which could predispose to the extrusion of debris and postoperative pain. Nevertheless, our results showed that, after 72 hours, postoperative pain was higher in teeth without periradicular lesion (*P* = 0.027). Marshall and Liesinger (65) observed that patients with periapical lesions that could not be detected by radiograph had more postoperative pain than those who with such lesions detected by radiograph. Ng *et al*. (33) found that postoperative pain was felt less in teeth having large periapical lesions (> 3 mm), compared with teeth having smaller or no periapical lesions. Genet *et al*. (66) found that postoperative pain was greater in teeth with periapical radiolucency greater than 5 mm in diameter. A periapical lesion from an endodontic infection might exist without being visible in the radiograph (67). It can be detected radiographically only when it attains nearly 30%–50% of the bone mineral loss (68,69). Other conditions, such as apical morphologic variations, the surrounding bone density, x-ray angulations and radiographic contrast also influence radiographic interpretation (70). These factors may explain the conflicting results of the studies cited above (33,65,66), and also the present findings.

Some studies showed that postoperative pain was more frequent and greater in posterior teeth (31,33,52). This could be attributed to their more complex root canal anatomy (54). Conversely, other studies pointed out that postoperative pain was not influenced by tooth type (37,71). In this research, tooth type also did not influence postoperative pain. As mentioned previously, an experienced operator (R.M.) performed all the treatments. This may have decreased the impact of the root canal anatomy, thus contributing to the low incidence and levels of postoperative pain.

The main limitation of the present study was the use of a single group. Analyses comparing different research results should be performed with caution, because of the potential methodological differences among them. Future studies should be carried out to assess postoperative pain after endodontic treatments performed by oscillatory versus other types of kinematics.

## Conclusions

The incidence and levels of postoperative pain after performing endodontic treatments in necrotic teeth submitted to LAP using oscillatory kinematics were low and not influenced by age and tooth type. When postoperative pain occurred, it tended to decrease over time.
